# Selective Interaction of Sugarcane eIF4E with VPgs from Sugarcane Mosaic Pathogens

**DOI:** 10.3390/v13030518

**Published:** 2021-03-22

**Authors:** Zongtao Yang, Meng Dong, Guangyuan Cheng, Shuxian Liu, Hai Zhang, Heyang Shang, Yingshuan Zhou, Guoqiang Huang, Muqing Zhang, Fengji Wang, Jingsheng Xu

**Affiliations:** 1 National Engineering Research Center for Sugarcane & Key Laboratory of Sugarcane Biology and Genetic Breeding, Ministry of Agriculture and Rural Affairs, Fujian Agriculture and Forestry University, Fuzhou 350002, China; YZT@fafu.edu.cn (Z.Y.); dongm6174@163.com (M.D.); chengguangyuanfafu@163.com (G.C.); liushuxian1010@163.com (S.L.); zhanghai940410@163.com (H.Z.); heyangshang@hotmail.com (H.S.); zhouyingshuan123@126.com (Y.Z.); hgq94@163.com (G.H.); 2 Key Laboratory of Ministry of Education for Genetics, Breeding and Multiple Utilization of Crops, College of Crop Science, Fujian Agriculture and Forestry University, Jinshan, Fuzhou 350002, China; 3 State Key Laboratory for Conservation and Utilization of Agro-Bioresources, Guangxi Key Laboratory for Sugarcane Biology, Guangxi University, Nanning 530005, China; zmuqing@163.com

**Keywords:** eIF4E, VPg, SCMV, SrMV, SCSMV

## Abstract

Eukaryotic translation initiation factor 4E (eIF4E) plays a key role in the infection of potyviruses in susceptible plants by interacting with viral genome-linked protein (VPg). Sugarcane (*Saccharum* spp.) production is threatened by mosaic disease caused by *Sugarcane mosaic virus* (SCMV), *Sorghum mosaic virus* (SrMV), and *Sugarcane streak mosaic virus* (SCSMV). In this study, two *eIF4Es* and their isoform *eIF(iso)4E* and 4E-binding protein coding genes were cloned from sugarcane cultivar *ROC22* and designated *SceIF4Ea*, *SceIF4Eb*, *SceIF(iso)4E,* and *ScnCBP*, respectively. Real-time quantitative PCR analysis showed different expression profiles of these four genes upon SCMV challenge. A subcellular localization assay showed that SceIF4Ea, SceIF4Eb, SceIF(iso)4E, and ScnCBP were distributed in the nucleus and cytoplasm. Yeast two-hybrid (Y2H) and bimolecular fluorescence complementation (BiFC) assays showed that SceIF4Ea/b and SceIF(iso)4E were selectively employed by different sugarcane mosaic pathogens, i.e., SCMV-VPg interacted with SceIF4Ea/b and SceIF(iso)4E, SrMV-VPg interacted with both SceIF4Eb and SceIF(iso)4E, and SCSMV-VPg interacted only with SceIF(iso)4E. Intriguingly, the BiFC assays, but not the Y2H assays, showed that ScnCBP interacted with the VPgs of SCMV, SrMV, and SCSMV. Competitive interaction assays showed that SCMV-VPg, SrMV-VPg, and SCMV-VPg did not compete with each other to interact with SceIF(iso)4E, and SceIF(iso)4E competed with SceIF4Eb to interact with SrMV-VPg but not SCMV-VPg. This study sheds light on the molecular mechanism of sugarcane mosaic pathogen infection of sugarcane plants and benefits sugarcane breeding against the sugarcane mosaic disease.

## 1. Introduction

Sugarcane (*Saccharum* sp.) is the most important sugar and energy crop worldwide [1,2,3]. Sugarcane mosaic disease is widespread in sugarcane-growing countries [4,5,6,7,8,9,10,11,12,13,14,15] and causes heavy yield losses [16,17]. In the 1920s, sugarcane mosaic disease almost collapsed the sugar industry in Argentina, Brazil, and Louisiana [18]. Although sugarcane mosaic disease is controlled by planting resistant sugarcane cultivars, it continues to be a threat to the sugarcane industry [4,15,17,19]. The main causal agents for the sugarcane mosaic disease are *Sugarcane mosaic virus* (SCMV), *Sorghum mosaic virus* (SrMV), and *Sugarcane streak mosaic virus* (SCSMV), which belong to the *Potyviridae* family, with SCMV and SrMV being members of the *Potyvirus* genus [20], whereas SCSMV is a member of *Poacevirus* genus [21,22,23]. Although SCMV, SrMV, and SCSMV belong to different genera in *Potyviridae*, they share very similar genomic structures. They all have a single-stranded positive-sense ~10 kb RNA genome encoding 2 polyproteins that are then proteolytically processed into 11 mature proteins: P1, helper component proteinase (HC-Pro), P3, P3N-PIPO, 6K1, cylindrical inclusion (CI), 6K2, viral genome-linked protein (VPg), proteinase domain of NIa (NIa-Pro), nuclear inclusion protein b (NIb), and CP [24,25,26,27,28,29,30,31,32].

In eukaryotic organisms, mRNA translation is predominantly cap dependent, and recognition of the cap structure (m^7^GpppN, where N is any nucleotide) at the 5′ terminus of mRNA is a key step in the cellular regulation of translation [33,34]. Translation initiation is a multistep process involving the assembly of a mRNA-protein complex by different eukaryotic translation initiation factors (eIFs). The binding of eIF4E to the cap structure of a mRNA is key for translation. Gaining access to the host translation machinery is essential for viruses in the *Potyviridae* family to establish infection in the host [25,35,36]. However, for the positive-sense RNA plant viruses in the *Potyviridae* and *Secoviridae* families and some members in the *Luteoviridae* family and *Sobemovirus* genus, there is no cap structure at the 5′ end of the genome RNA [25,37]. VPg serves as an analog of the cap structure by covalently linking to the 5′ end of virus sense genome RNA via a tyrosine or serine residue [38,39,40,41]. It has been well documented that the VPg of potyviruses interacts with the host cap-binding eIF4E or eIF(iso)4E proteins to initiate viral genome translation [25,35,37,42,43].

Silencing of *eIF4E*, *eIF(iso)4E* or *nCBP* contributes to mutant plant resistance to one or more species of viruses. In *Arabidopsis thaliana*, *eIF(iso)4E* mutant plants are resistant to *Turnip mosaic virus* (TuMV), *Tobacco etch virus* (TEV) or *Lettuce mosaic virus* (LMV) [44,45]. The *eIF4E*-mutant plants are resistant to *Clover yellow vein virus* (ClYVV) [46]. In pepper, the knockout mutation of *eIF4E* causes resistance to *Potato virus Y* (PVY) and TEV [47,48]. The mutant of lettuce *eIF4E* is resistant to *Lettuce mosaic virus* (LMV) [49]. In tomato, knockdown of *eIF4E1* confers resistance to both PVY and *Pepper mottle virus* (PepMoV) [50]. Further, knockdown of both the *eIF4E1* and *eIF4E2* genes confers broad-spectrum resistance against PVY, TEV, *Pepper mottle virus* (PepMoV), *Ecuadorian rocotto virus* (ERV), *Pepper severe mosaic virus* (PepSMV), *Pepper yellow mosaic virus* (PepYMV), and *Potato virus V* (PVV) in mutant tomato plants [51]. Furthermore, most of the mutant lines show no growth defects. A study of these mutants led to the discovery of the molecular nature of plant recessive resistance to plant viruses [42,52,53,54]. To date, 14 natural recessive resistance genes corresponding to *eIF4E* or its isoform *eIF(iso)4E* against plant viruses have been identified from diverse plant species [37,55,56].

The molecular mechanism of sugarcane mosaic pathogen infection in sugarcane is poorly understood [15,17,30,57,58,59] due to the highly complex sugarcane genome and difficulty of performing transformation [59,60]. In the present study, we cloned 4 *eIF4E* homologs obtained from sugarcane cultivar *ROC22* and designated them as *SceIF4Ea*, *SceIF4Eb*, *SceIF(iso)4E*, and *ScnCBP*. Their expression profiles upon the SCMV challenge were investigated by real-time quantitative PCR, and the interaction with SCMV-/SrMV-/SCSMV-VPg was individually explored by yeast two-hybrid (Y2H) and bimolecular fluorescence complementation (BiFC) assays. This study is helpful for understanding the molecular mechanism of SCMV, SrMV or SCSMV infection of sugarcane and instructive for the molecular breeding for sugarcane resistance to mosaic diseases.

## 2. Materials and Methods

### 2.1. Plant Materials and Viruses

SCMV, SrMV, and SCSMV isolates were provided by the Key Laboratory of Sugarcane Biology and Genetic Breeding, Ministry of Agriculture and Rural Affairs, P.R. China. Healthy seed canes were propagated from the axillary bud of cv. *ROC22* and exposed to a 14/10-h light/dark cycle at 28 °C in the greenhouse. *ROC22* plantlets were individually inoculated with SCMV at the 5–6 leaf stage, as previously described [59], and mock-inoculated *ROC22* plantlets with 0.01 M phosphate buffer (pH 7.0) were used as the control. The inoculated or mock inoculated leaves were sampled on 0, 1, 2, and 5 days post-inoculation. All plant materials were collected in triplicate, immediately frozen in liquid nitrogen, and then placed in a −80 °C refrigerator for RNA isolation. *Nicotiana benthamiana* plants were grown at 22 ± 0.5 °C and 70% relative humidity under a 16-h day/8-h night photoperiod in climate-controlled cabinets. Illumination of 90 μmol/s/m^2^ was generated by a fluorescent lamp.

### 2.2. RNA Extraction and Real-Time Quantitative PCR

Leaf samples were ground into powder in liquid nitrogen. One hundred milligrams of leaf powder was mixed with 1 mL of TriPure reagent (Roche, Basel, Switzerland). Total RNA was extracted according to the manufacturer’s instructions. The RNA concentration and quality were determined using an ND-1000 spectrophotometer (NanoVue Plus, GE, Chicago, IN, USA) and electrophoresis. First-strand cDNA was synthesized using a PrimeScript RT-PCR kit (TaKaRa, Dalian, China) according to the manufacturer’s instructions.

Real-time quantitative PCR (RT-qPCR) was carried out using the Fast Universal SYBR Green Master mix (ROX; Roche, Hercules, CA, USA) on an ABI7500 real-time PCR system (Applied Biosystems, Foster City, CA, USA). *GAPDH* [61] and *eEF-1α* were used as internal references [62], and three replicates were completed for each sample. A melting curve analysis was conducted to confirm the PCR specificity for each primer pair. The data were analyzed using the 2^−ΔΔCt^ method to determine the altered gene expression, and the results are shown as the mean of three biological replicates with the corresponding standard error.

### 2.3. Plasmid Construction

All primers used for plasmid construction are listed in Supplemental Table S1. For the Y2H experiments, the yeast two-hybrid vectors and all DNA fragments were individually ligated via *Sfi*I sites. *SCMV-VPg*, *SrMV-VPg*, *SCSMV-VPg*, and *ScnCBP* were individually cloned into the bait vector pGBKT7. *SceIF4Ea*, *SceIF4Eb*, *SceIF(iso)4E,* and *ScnCBP* were individually cloned into the acquired vector pGADT7. For the pBridge vector construction, *SCMV-/SrMV-/SCSMV-VPg* was individually inserted into multiple clone site 1 (MCS1) using restrictive endonuclease *Xma*I, while *SCMV-/SrMV-/SCSMV-VPg*, *SceIF4Eb*, and *SceIF(iso)4E* were individually inserted into MCS2 using restrictive endonuclease *Not*I.

For the transient protein expression and BiFC assays, all plasmids were generated using the gateway technology, as described by Cheng et al. [31]. The resulting respective DNA fragments were purified and cloned into a pDONR221 entry vector using the BP reaction according to the manufacturer’s instructions. The pDONR221 vector containing the target gene was recombined into the destination vector pEarleyGate201-YN, pEarleyGate202-YC, and pEarleyGate101 using the LR reaction following the manufacturer’s instructions to yield C-terminal YN, C-terminal YC, and C-terminal YFP fusion constructs, respectively. All plasmids generated in this study were verified by sequencing.

### 2.4. Protein Interaction as Determined by Y2H and BiFC Assays

For the Y2H assay, the Matchmaker Gold Yeast Two-Hybrid System (Clontech, Mountain View, CA, USA) was used according to the manufacturer’s protocols. The activation domain (AD) vector pGADT7 and the GAL4 DNA-binding domain (BD) vector pGBKT7 were used. The prey vector pGADT7 and bait vector pGBKT7 harboring the genes to be tested were co-transformed pairwise into the yeast (*Saccharomyces cerevisiae*) strain AH109. SD/-Trp/-Leu (DDO) agar plates and SD/-Trp/-Leu/-His/-Ade (QDO) agar plates were used for the yeast cultures. Cells were spread on DDO plates and incubated at 30 °C for 3–5 days after transformation. Colonies grown on DDO plates were suspended in a DDO liquid medium to an OD_600_ of 0.6. A 10× dilution series of 5 μL aliquots of co-transformed AH109, which were spotted onto DDO or QDO agar plates supplemented with 5-Bromo-4-chloro-3-indolyl-α-D-galactopyranoside (X-α-Gal) to test the expression of the MEL1 marker. The plates were incubated at 30 °C for 3–5 days. Additionally, pGADT7-T and pGBKT7-53 interacted with the Y2H assays and were used as positive controls. Moreover, pGADT7-T and pGBKT7-Lam did not form complexes and were used as negative controls. For the competitive Y2H assays, co-transformed yeast cells were harvested from DDO agar plates and shaken in YDPA at 30 °C for 12 h. Then, the OD_600_ was adjusted to 1.0 and the culture was diluted at a ratio of 1:1000 in a QDO liquid medium at 30 °C for 12 h. The absorbance was measured at OD_600_ to quantify the interaction strength.

For the BiFC assay, two YFP fusion constructs were transformed into *A. tumefaciens* GV3101. Agrobacterium cultures were grown to an optical density of 1.0 at 600 nm (OD_600_). Equal volumes of each culture were mixed and infiltrated in *N. benthamiana* leaves using needleless syringes. The agroinfiltrated plants were maintained under normal growth conditions for 48 to 72 h.

### 2.5. Transient Expression

The plasmids were transformed into *A. tumefaciens* GV3101. The transformed GV3101 was agroinfiltrated into the leaves of *N. benthamiana* using a needleless syringe. Agrobacteria were cultured overnight in a Luria–Bertani medium containing the appropriate antibiotics and collected by centrifugation. Then, the agrobacteria were resuspended in 10 mM MgCl_2_ containing 100 mM acetosyringone. The culture was incubated for 2–3 h at room temperature and then diluted to an OD_600_ of 0.2–0.5. *N. benthamiana* plants were agroinfiltrated with agrobacterial cultures, and the agroinfiltrated plants were maintained under normal growth conditions for 48 to 72 h. A diamidine phenylindole (DAPI) stock solution (1.0 mg/mL) was prepared and stored at −20 °C. A DAPI stock solution was diluted 1:1000 in double-distilled H_2_O to prepare the working solution. The *N. benthamiana* leaves were incubated in the working solution for 10–20 min at room temperature to label the nucleus.

### 2.6. Confocal Microscopy

Agroinfiltrated leaf sections were imaged at room temperature using a Leica SP8 X inverted confocal microscope with an argon laser (Leica, Wetzlar, Germany). DAPI was excited at 340–488 nm, and the emitted light was captured at 505–555 nm. GFP was excited at 488 nm, and the emitted light was captured at 505–555 nm. YFP was excited at 514 nm, and the emitted light was captured at 530–590 nm. Images were captured digitally and processed using the Leica Application Suite Advanced Fluorescence Lite software (LAS AF; version 2.6.3; build 8173).

### 2.7. Multiple Sequence Alignment

The amino acid sequences of eIF4Es and nCBPs from other plant species were retrieved from the NCBI (https://www.ncbi.nlm.nih.gov/, accessed on 28 February 2021). The DNAman 6.0 software and ClustalW2 program were used for the visualization and editing of the aligned sequences. The MEGA 7.0 software was used to construct the phylogenetic tree.

## 3. Results

### 3.1. Cloning of SceIF4Ea, SceIF4Eb, SceIF(iso)4E, and ScnCBP from Sugarcane

**Figure 1:**
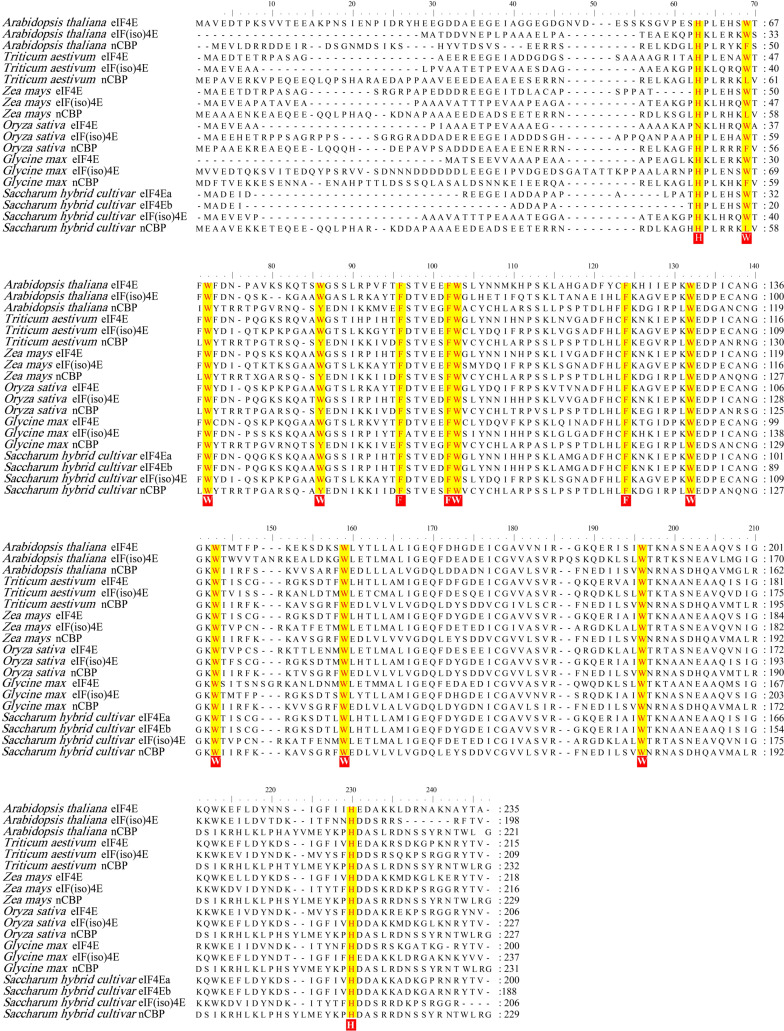
Alignment of the amino acid sequences of the eIF4E, eIF(iso)4E, and nCBP from several plant species. The accession numbers of the selected amino acid sequences were *Arabidopsis thaliana*: eIF4E (AT4G18040), eIF(iso)4E (AT5G35620), nCBP (AT5G18110); *Triticum aestivum*: eIF4E (CAA78262), eIF(iso)4E (AAA34296), nCBP (XM.037569593); *Zea mays*: eIF4E (AF076954), eIF(iso)4E (CD527566), nCBP (EU959765); *Oryza sativa*: eIF4E (U34597), eIF(iso)4E (U34598), nCBP (CF328046); *Glycine max*: eIF4E (BI785638), eIF(iso)4E (BG045933), nCBP (BE474869); *Saccharum* spp. hybrid *ROC22*: SceIF4Ea (MW547070), SceIF4Eb (MW547071), SceIF(iso)4E (MW547073), ScnCBP (KX757019). Conserved Trp, Phe, and His residues were yellow shaded: H(X5)W(X2)W(X8–12)W-(X9)F(X5)FW(X20)F(X7)W(X10)W(X9–12)W(X34–35)W(X31–33)H. Numbers to the right of the sequences indicate the positions of residues from the N-terminal Met.

Four eIF4E homologs were cloned from sugarcane cultivar *ROC22* based on homologous cloning. The ORFs of *SceIF4Ea*, *SceIF4Eb*, *SceIF(iso)4E*, and *ScnCBP* are 603, 567, 621, and 690 bp, respectively, with the corresponding GenBank accession numbers of MW547070, MW547071, MW547072, and KX757019. SceIF4Ea is almost identical to SceIF4Eb, with only a few amino acid differences in the N-terminus. The deduced amino acid sequences of the four SceIF4E homologs were compared with homologs from *Arabidopsis thaliana*, *Triticum aestivum*, *Zea mays*, *Oryza sativa,* and *Glycine max*. The results showed that the amino acid residues Trp, Phe, and His in the four SceIF4E homologs showed the distinctive pattern of eIF4E family: H(x_5_)W(x_2_)W(x_8–12_)W(x_9_)F(x_5_)FW(x_20_)F(x_7_)W(x_10_)W(x_12–15_)W(x_34–35_)W(x_31–33_)H (X stands for an arbitrary amino acid) (Figure 1), as described by Joshi et al. [63].

**Figure 2:**
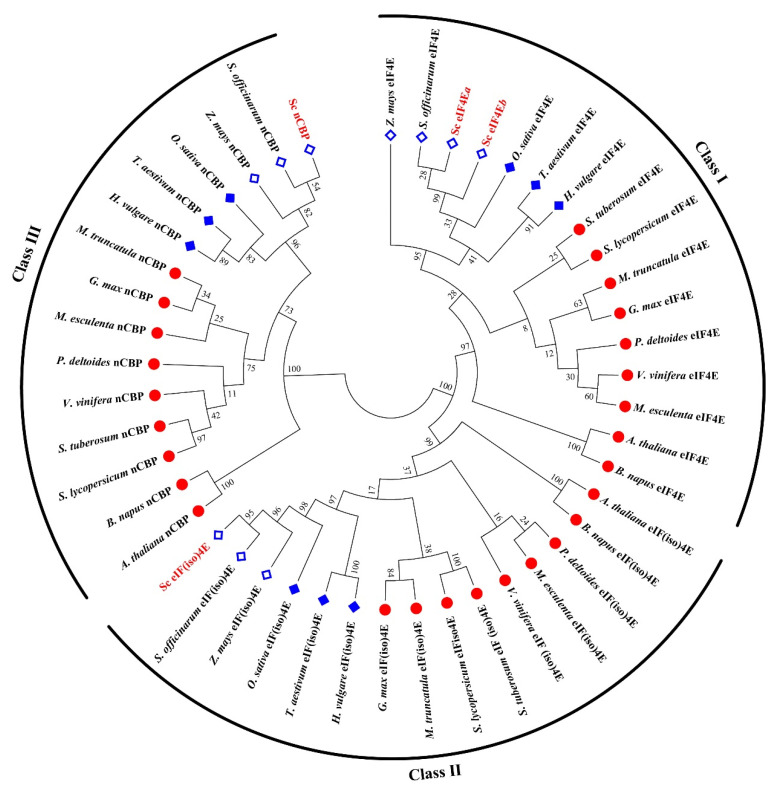
Phylogenetic tree analysis of eIF4E, eIF(iso)4E, and nCBP proteins from different plant species. Protein sequences were retrieved from NCBI and the accession numbers are as follows. *Arabidopsis thaliana*: eIF4E (AT4G18040), eIF(iso)4E (AT5G35620), nCBP (AT5G18110); *Triticum aestivum*: eIF4E (CAA78262), eIF(iso)4E (AAA34296), nCBP (XM.037569593); *Zea mays*: eIF4E (AF076954), eIF(iso)4E (CD527566), nCBP (EU959765); *Vitis vinifera*: eIF4E (BM437772), eIF(iso)4E (CB918946), nCBP (BM437090); *Solanum tuberosum*: eIF4E (BI178346), eIF(iso)4E (BG598942), nCBP (CK269484); *Saccharum officinarum*: eIF4E (CA248584), eIF(iso)4E (CA120131), nCBP (CA241493); *Populus deltoides*: eIF4E (CX176909), eIF(iso)4E (CV130917), nCBP (CX173803); *Oryza sativa*: eIF4E (U34597), eIF(iso)4E (U34598), nCBP (CF328046); *Medicago truncatula*: eIF4E (AJ502732), eIF(iso)4E (AL378243), nCBP (AL381239); *Hordeum vulgare*: eIF4E (CA008276), eIF(iso)4E (BU987334), nCBP (BQ467551); *Glycine max*: eIF4E (BI785638), eIF(iso)4E (BG045933), nCBP (BE474869); *Brassica napus*: eIF4E (CD814531), eIF(iso)4E (CD826731), nCBP (CD842187); *Solanum lycopersicum*: eIF4E (Solyc03g005870), eIF(iso)4E (Solyc09g090580), nCBP (Solyc10g080660); *Manihot esculenta*: eIF4E (MANES.17G063100), eIF(iso)4E (MANES.03G160000), nCBP (MANES.09G140300); *Saccharum* spp. hybrid *ROC22*: SceIF4Ea (MW547070), SceIF4Eb (MW547071), SceIF(iso)4E (MW547073), ScnCBP (KX757019), which were highlighted by the “leaves” in red. The red circle indicated the dicotyledons. The blue diamond indicated the monocotyledons with the hollow diamond for C4 plants, while the solid core of diamond for C3 plants.

The phylogenetic tree was constructed using the deduced amino acid sequences of the four SceIF4E homologs and their corresponding homologs from different plant species (Figure 2). The phylogenetic analysis revealed that the four SceIF4E homologs were clustered into three distinct clades with SceIF4Ea and SceIF4Eb in Class I, SceIF(iso)4E in Class II, and ScnCBP in Class III. In each clade, two obvious subgroups were identified representing sequences from monocots or dicots, and different groups were formed by C4 and C3 monocot plants (Figure 2).

### 3.2. Expression Profiles of SceIF4Ea, SceIF4Eb, SceIF(iso)4E, and ScnCBP Upon the SCMV Challenge

**Figure 3:**
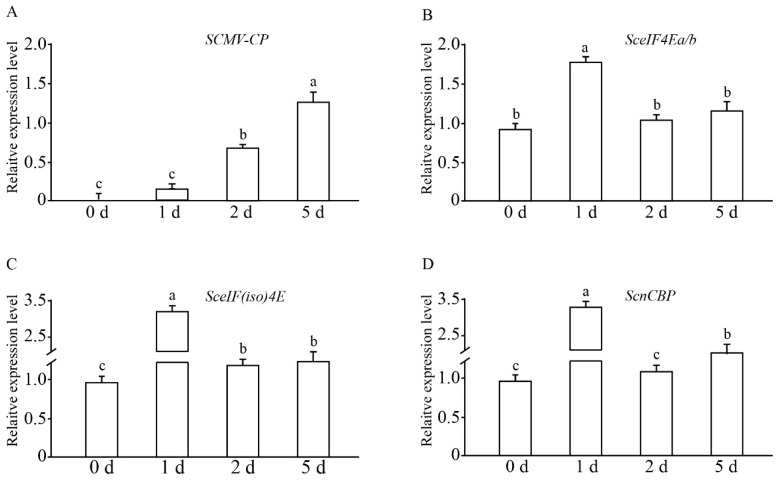
The expression profiles of *SceIF4Ea/b*, *SceIF(iso)4E,* and *ScnCBP* of sugarcane cultivar *ROC22* challenged by *Sugarcane mosaic virus* (SCMV). Leaves of *ROC22* plantlets were inoculated with SCMV and sampled at different time points. Mock inoculated plants with 0.01 M phosphate buffer (pH 7.0) were used as the negative controls. The Y axes indicates the relative expression of *SCMV-CP* (**A**), *SceIF4Ea/b* (**B**), *SceIF(iso)4E* (**C**), and *ScnCBP* (**D**) at 0, 1, 2, and 5 days post-inoculation. The X axes indicates the time point of material collection. Error bars indicate SD (*n* = 3), a, b, and c indicate significance at the corresponding time points, *t*-test, *p* <0.05. Results were representative of three independent experiments.

The transcriptional expression profile of the four SceIF4E homologs in sugarcane cultivar *ROC22* plantlets challenged by SCMV was investigated by RT-qPCR. The expression of the *CP* gene of SCMV increased with the infection progression (Figure 3), indicating the replication of the SCMV genome and successful infection of SCMV. As the high similarity of *SceIF4Ea* and *SceIF4Eb* made differences difficult to be distinguished by RT-qPCR, their expression profiles were combined using one pair of primers. The results showed that the expression levels of *SceIF4Ea/b*, *SceIF(iso)4E,* and *ScnCBP* were upregulated upon infection and then downregulated to the level of the control or slightly higher than the control level (Figure 3), which is similar to the soybean response to the *Soybean mosaic virus* infection [54].

### 3.3. Subcellular Localization of SceIF4Ea, SceIF4Eb, SceIF(iso)4E, and ScnCBP

**Figure 4:**
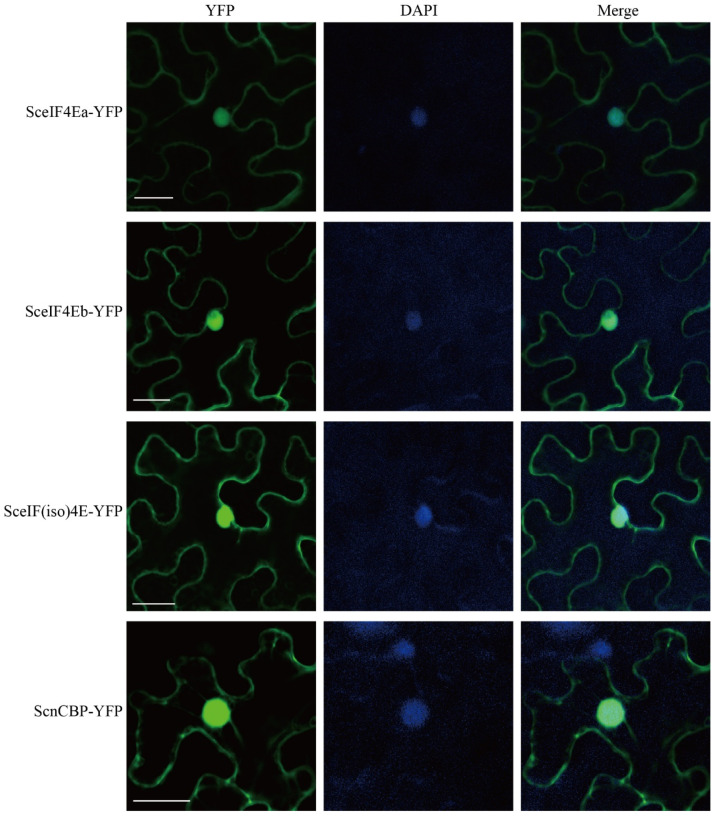
Subcellular localization of SceIF4Ea, SceIF4Eb, SceIF(iso)4E, and ScnCBP in *Nicotiana benthamiana* leaf epidermal cells. Fluorescence of YFP or YFP fusion proteins was detected by 48 h post agroinfiltration. The nucleus was displayed by diamidine phenylindole (DAPI) staining. Bars = 25 μm.

To determine the subcellular localization of the four SceIF4E homologs, SceIF4Ea-YFP, SceIF4Eb-YFP, SceIF(iso)4E-YFP, and ScnCBP-YFP were individually expressed in *N. benthamiana* leaves by agroinfiltration. The leaves were infiltrated with DAPI and imaged by confocal laser scanning microscopy. YFP fluorescence was distributed in the cytoplasm and overlapped the fluorescence of DAPI in the nucleus (Figure 4), indicating that SceIF4Ea, SceIF4Eb, SceIF(iso)4E, and ScnCBP localized to the cytoplasm and nucleus.

### 3.4. Interaction of SceIF4Ea, SceIF4Eb, SceIF(iso)4E, and ScnCBP with SCMV-/SrMV-/SCSMV-VPg

**Figure 5:**
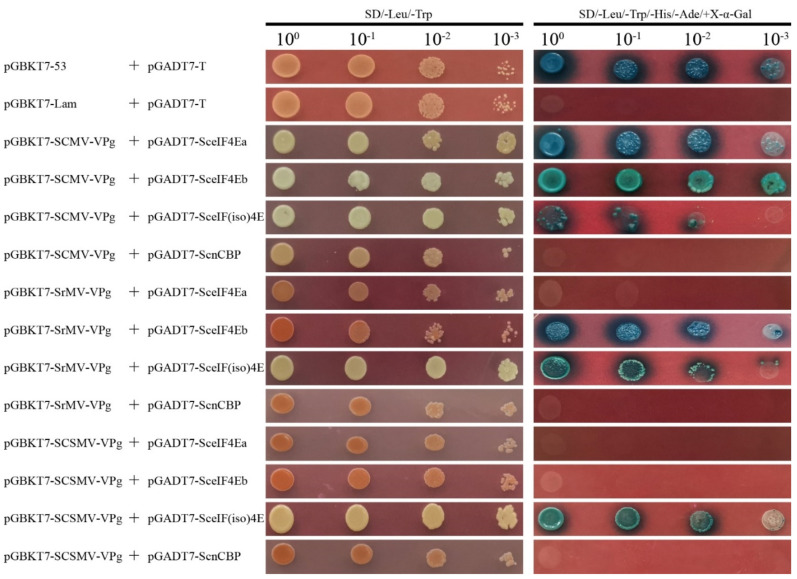
Investigation of protein interaction by yeast two-hybrid assays. The coding sequences of SceIF4Ea, SceIF4Eb, SceIF(iso)4E, and ScnCBP were individually infused into the prey vector pGADT7 and pairwise co-transformed with the vector pGBKT7-SCMV-VPg, pGBKT7-SrMV-VPg, pGBKT7-SCSMV-VPg into the yeast *AH109* cells in a 10× dilution series of 10-µL aliquots, which were then plated on a non-selective medium SD/-Leu/-Trp or quadruple dropout medium SD/-Leu/-Trp/-His/-Ade supplemented with X-α-Gal. Yeast cells co-transformed with pGBKT7-53 and pGADT7-T were used as a positive control, pGBKT7-Lam and pGADT7-T were used as a negative control.

Y2H based on GAL4 was applied to determine the interaction of SCMV-/SrMV-/SCSMV-VPg with the four SceIF4E homologs. The bait vectors pGBKT7-SCMV-VPg, pGBKT7-SrMV-VPg, and pGBKT7-SCSMV-VPg were individually co-transformed with the prey vectors pGADT7-SceIF4Ea, pGADT7-SceIF4Eb, pGADT7-SceIF(iso)4E, and pGADT7-ScnCBP into the *Saccharomyces cerevisiae* strain AH109. Similar to the yeast cells co-transformed with the positive control plasmids pGBKT7-53 and pGADT7-T, the yeast cells transformed with the combination of pGBKT7-SCMV-VPg and pGADT7-SceIF4Ea, pGBKT7-SCMV-VPg and pGADT7-SceIF4Eb, pGBKT7-SCMV-VPg and pGADT7-SceIF(iso)4E, pGBKT7-SrMV-VPg and pGADT7-SceIF4Eb, pGBKT7-SrMV-VPg and pGADT7-SceIF(iso)4E, pGBKT7-SCSMV-VPg and pGADT7-SceIF(iso)4E produced blue colonies on the DDO culture medium and blue colonies on the QDO culture medium supplemented with X-α-Gal, while other combinations showed no interaction, similar to the negative control plasmids pGBKT7-Lam and pGADT7-T (Figure 5). However, the yeast cells that co-transformed with pGBKT7-SCMV-VPg and pGADT7-SceIF(iso)4E did not grow well, indicating the weak interaction between SCMV-VPg and SceIF(iso)4E (Figure 5).

**Figure 6:**
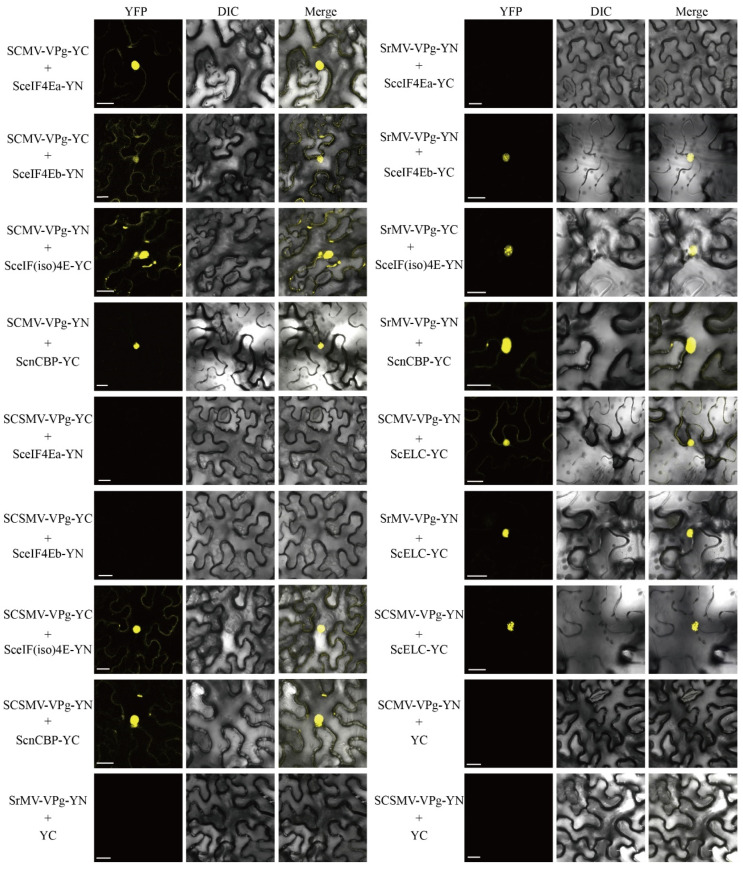
Investigation of protein interaction by bimolecular fluorescence complementation assays. Agrobacteria harboring YC/YN fusion proteins were co-infiltrated into *Nicotiana benthamiana* leaves, respectively. The leaf epidermal cells pairwise co-transformed with SCMV-VPg-YN, SrMV-VPg-YN or SCSMV-VPg-YN and ScELC-YC were used as positive controls, while SCMV-VPg-YN, SrMV-VPg-YN or SCSMV-VPg-YN and YC were used as negative controls. The images were captured at 48 h post agroinfiltration. Bars = 25 μm.

To further test the interactions of SCMV-/SrMV-/SCSMV-VPg with the four SceIF4E homologs *in planta*, BiFC assays were conducted with *N. benthamiana* leaves. The fusion constructs SCMV-VPg-YC, SrMV-VPg-YC, and SCSMV-VPg-YC were individually cotransformed with SceIF4Ea-YN, SceIF4Eb-YN, SceIF(iso)4E-YN or ScnCBP-YN into *Agrobacterium tumefaciens* GV3101 and then agroinfiltrated into *N. benthamiana* leaves. Within 48 hpa, the yellow fluorescence of YFP was observed by confocal microscopy. The results of the BiFC assays confirmed the interaction between SCMV-/SrMV-/SCSMV-VPg and SceIF4Ea, SceIF4Eb, SceIF(iso)4E or ScnCBP, as demonstrated by the Y2H assays, with the exception that ScnCBP interacted with SCMV-/SrMV-/SCSMV-VPg (Figure 6). As expected, interactions with the positive controls SCMV-VPg-YN and ScELC-YC were observed [57], while the negative controls emitted no fluorescence signals (Figure 6).

### 3.5. Competitive Interaction of SceIF4Ea, SceIF4Eb, SceIF(iso)4E with SCMV-/SrMV-/SCSMV-VPg

**Figure 7:**
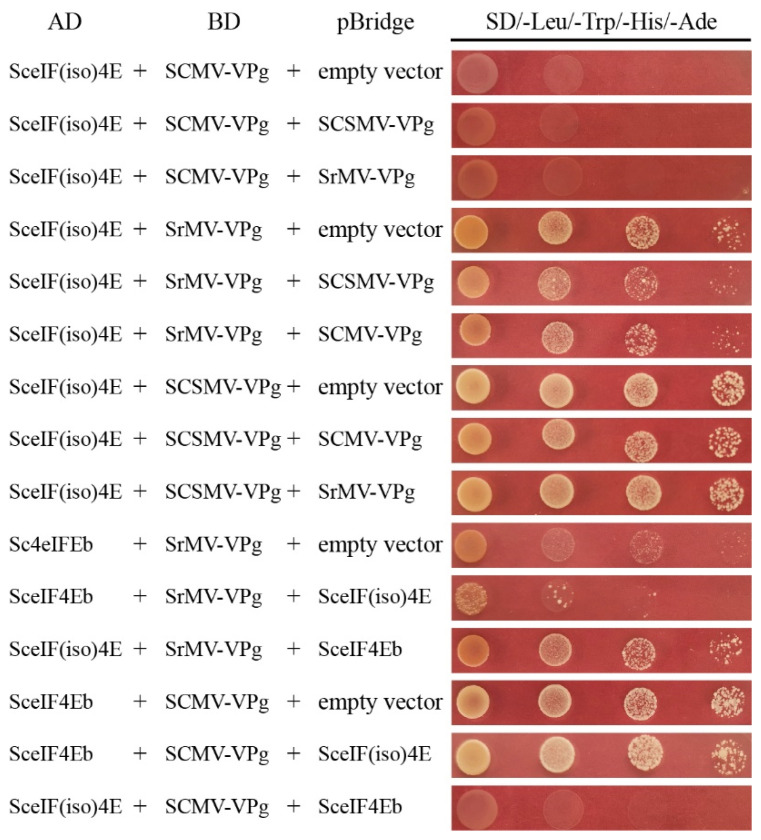
Competitive yeast two-hybrid assays. SCMV-VPg, SrMV-VPg, SCSMV-VPg, eIF4Eb, eIF(iso)4E were used as the competitor to test the interaction of SCMV-VPg, SrMV-VPg, SCSMV-VPg with eIF4Eb, eIF(iso)4E, respectively. The competitor was co-transformed with the paired interacting proteins fused with the activation domain (AD) or DNA-binding domain (BD) into the yeast AH109 cells in a 10× dilution series of 10-µL aliquots, which were then plated on the quadruple dropout medium SD/-Leu/-Trp/-His/-Ade.

Competitive Y2H assays were performed to investigate the interaction of SceIF4Ea, SceIF4Eb, and SceIF(iso)4E with SCMV-/SrMV-/SCSMV-VPg. Interacting proteins were inserted into the bridge vector and used as competitors. The interacting proteins were constructed into AD or BD vectors and pairwise co-transformed with the competitor or an empty pBridge vector as a negative control into the *S. cerevisiae* strain AH109 cultured on a QDO agar medium. Yeast cells co-transformed with the competitor or an empty pBridge vector were cultured in a QDO liquid medium. The QDO liquid medium was also used to quantify the growth of the transformed yeast cells by monitoring the OD_600_ value. As SceIF(iso)4E interacted with SCMV-VPg, SrMV-VPg, and SCSMV-VPg, each VPg was expressed via a pBridge vector to investigate the interaction of other VPgs with SceIF(iso)4E. Compared with the controls, the yeast cells showed no obvious difference, indicating that SCMV-VPg, SrMV-VPg, and SCSMV-VPg did not compete with each other for the interaction with SceIF(iso)4E (Figure 7), as demonstrated by QDO liquid medium culture assays (Figure S1a–c). As SrMV-VPg interacted with SceIF4Eb or SceIF(iso)4E, SceIF4Eb or SceIF(iso)4E was individually constructed into a pBridge vector to investigate the interaction of SrMV-VPg with eIF4Eb or SceIF(iso)4E, respectively. The results showed that SceIF(iso)4E interfered with the interaction between SceIF4Eb and SrMV-VPg, while SceIF4Eb did not interfere with the interaction between SceIF(iso)4E and SrMV-VPg (Figure 7; Figure S1d), indicating that SceIF(iso)4E is preferentially utilized by SrMV. For the interaction of SCMV-VPg with SceIF4Eb or SceIF(iso)4E, the results showed that SceIF4Eb did not interfere with the interaction between SCMV-VPg and SceIF(iso)4E, while SceIF(iso)4E did not interfere with the interaction between SCMV-VPg and SceIF4Eb, which was confirmed by QDO liquid medium culture assays (Figure 7; Figure S1e).

## 4. Discussion

In the present study, the coding genes of SceIF4Ea, SceIF4Eb, SceIF(iso)4E, and ScnCBP were cloned from susceptible sugarcane cultivar *ROC22,* and their interaction with the VPgs of SCMV, SrMV or SCSMV was investigated. This study indicated that SceIF4Ea, SceIF4Eb, and SceIF(iso)4E were selectively employed by SCMV, SrMV or SCSMV. Two eIF4Es were identified, i.e., SceIF4Ea and SceIF4Eb, whose sequences were similar, with only few amino acid residues differences at the N-terminus (Figure 2). Intriguingly, SceIF4Ea and SceIF4Eb interacted with SCMV-VPg, whereas SceIF4Eb interacted with SrMV-VPg, but SceIF4Ea did not (Figure 5 and Figure 6), which indicated that the N-terminus of eIF4E is involved in the interaction with potyviral VPg. It might deepen the understanding of the molecular mechanism of VPg interaction with eIF4E to identify the key residue in the N-terminus of SceIF4Eb that mediates the interaction with SrMV-VPg.

ScnCBP did not interact with the VPgs of SCMV, SrMV or SCSMV in the Y2H assays, but the BiFC assays with the *N. benthamiana* leaves yielded positive results (Figure 5). In *A. thaliana*, nCBP showed affinity to the cap structure comparable to that shown by eIF(iso)4E [64]. The cap binding complex comprises many components [65,66]. Considering that *ScnCBP* was upregulated upon SCMV infection (Figure 3), we speculate that other cap binding components mediate the interaction of ScnCBP with the VPgs of SCMV, SrMV or SCSMV in vivo. The nCBP mediated recessive resistance against viruses in the *Alphaflexiviridae* and *Betaflexiviridae* families [67]. Simultaneous CRISPR/Cas9-mediated editing of two nCBP alleles reduced the cassava brown streak disease symptom severity and incidence caused by *Cassava brown streak virus* and *Ugandan cassava brown streak virus* in the *Potyviridae* family [68]. Therefore, it is reasonable to conclude that ScnCBP plays roles in the response to the mosaic virus infection of sugarcane.

SCMV-VPg, SrMV-VPg, and SCSMV-VPg interacted and did not compete with each other for the interaction with SceIF(iso)4E (Figure 5, Figure 6 and Figure 7), indicating that SceIF(iso)4E was extensively employed by SCMV, SrMV, and SCSMV. Specifically, SCSMV-VPg was found to selectively interact with SceIF(iso)4E (Figure 5 and Figure 6), making *SceIF(iso)4E* an eminent target for the molecular breeding of sugarcane plants with resistance to SCSMV. As SceIF(iso)4E was preferentially employed by SrMV (Figure 7 and Figure S1), mutation of *SceIF(iso)4E* might force SrMV to utilize SceIF4Eb with suboptimal binding affinity, thereby attenuating mosaic disease progression caused by SrMV.

Growing virus-resistant sugarcane cultivars are the best way to prevent severe losses of yield and quality [4]. However, it is difficult to combine eminent agricultural traits with mosaic resistance due to the complicated sugarcane genome [60]. With the development of RNAi and the increasing regeneration efficiency of sugarcane calli, the *CP* gene was utilized to create resistant sugarcane germplasm against SCMV or SrMV [17,69]. However, the *CP* genes varied greatly in sequence [70]. Genetically modified sugarcane plants that transformed with the *CP* gene from SrMV-SCH were resistant to SrMV-SCH and the closely related SrMV-SCI and SrMV-SCM strains (95% nucleotide similarity) but not to the more distantly related SCMV strain D (75% nucleotide similarity) [69]. Therefore, the resistance derived from pathogens might make it difficult to create broadly resistant germplasms. As there are numerous successful reports of potyviruses management obtained by the mutation of eIF4E or its isoform [44,45,46,47,48,49,50,51] and the identification of natural recessive resistance genes against potyviruses [37,55,56,67], mutation of *SceIF(iso)4E* may confer to sugarcane plants resistance to SCSMV, while simultaneous mutation of *SceIF4Eb* and *SceIF(iso)4E* may confer to sugarcane plants broad resistance to both SrMV and SCSMV. However, this method should be tested by comprehensive experiments, as a recent study by Zafirov et al. showed that *eIF4E1*-knockout *A. thaliana* mutants were resistant to *Clover yellow vein virus* (ClYVV) but hypersusceptible to TuMV [71]. Zafirov et al. suggested that gene editing by CRISPR/Cas9 is superior to gene knockout [71,72,73]. Therefore, further experiments should be conducted to investigate the key amino acid residues that mediate the interaction of SceIF4Ea, SceIF4Eb, SceIF(iso)4E or ScnCBP with SCMV-VPg, SrMV-VPg or SCSMV-VPg.

## Data Availability

The data that support the findings of this study are available from the corresponding author upon reasonable request.

## Supplementary Materials

The following are available online at https://www.mdpi.com/1999-4915/13/3/518/s1, Figure S1: Yeast growth assay, Table S1: Primers used in this study.
