# Supplementary material for: Selective Interaction of Sugarcane eIF4E with VPgs from Sugarcane Mosaic Pathogens
Source: Viruses. 2021 Mar 22;13(3):518. doi: 10.3390/v13030518 (PMC8005120; doi:10.3390/v13030518)
Supplement: Supplementary file 1 [file viruses-13-00518-s001.pdf]

Supplementary Materials:

Figure S1:

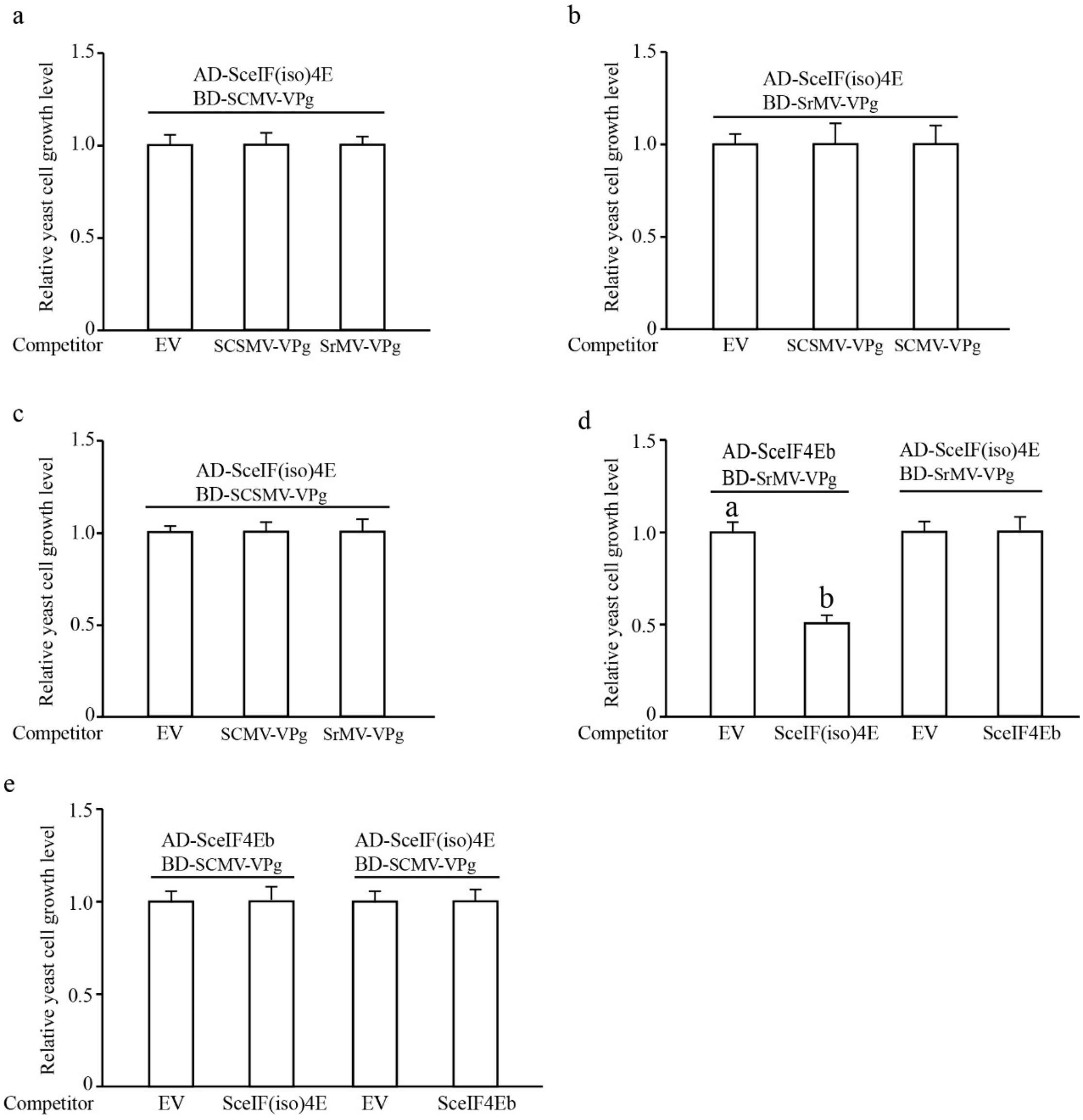

Figure S1. Yeast growth assay. SCMV-VPg, SrMV-VPg, SCSMV-VPg, eIF4Eb, eIF(iso)4E were used as the competitor to test the interaction of SCMV-VPg, SrMV-VPg, SCSMV-VPg with eIF4Eb, eIF(iso)4E, respectively. The competitor were co-transformed with the paired interacting proteins fused with activation domain (AD) or DNA-binding domain (BD) into the yeast *AH109* cells, which were then cultured in the liquid quadruple dropout medium SD/-Leu/-Trp/-His/-Ade at 30 °C for 12 h. Then, the value of OD_{600} was measured to quantify the interaction strength (Student's *t*-test, $P<0.05$). EV: Empty vector.

Table S1: Primers used in this study.

**Name**	**Sequence (5′ - 3′)**
**Primers used to clone gene sequences**	
SceIF4Ea-F	ATGGCCGACGAGATCGACAG
SceIF4Ea-R	TCAAACCGTGTAACGGTTCCTC
SceIF4Eb-F	ATGGCCGACGAGATCGCCGATGAC
SceIF4Eb-R	TCAAACCGTGTAACGGTTCCTC
SceIF(iso)4E-F	ATGGCGGAGGTCGAGGTTCC

SceIF(iso)4E-R	TTACCGTCCGCCTCTGCTCG
ScnCBP-F	ATGGAGGCGGCGGTGGAGAA
ScnCBP-R	TTATCCTCTCAGCCAAGTGTTC
SCMV-VPg-F	ATGGGAAAGAACAAGCGCAGCAG
SCMV-VPg-R	GAGACAGGTGTCGCGCACGA
SrMV-VPg-F	ATGGGTAAAAACAAGAGGAGTAG
SrMV-VPg-R	TTCATGTTCTACTTCAACCT
SCSMV-VPg-F	ATGGGGAAGAAGCGTCGAACTC
SCSMV-VPg-R	CTCACTAGTCAACGCCTGTTG
**Primers used to construct subcellular localization and BiFC vectors**	
attB-SceIF4Ea-F	GGGGACAAGTTTGTACAAAAAAGCAGGCTTCATGATGGCCGACG AGATCGACAG
attB-SceIF4Ea-R	GGGGACCACTTTGTACAAGAAAGCTGGGTCAACCGTGTAACGGTT CC
attB-SceIF4Eb-F	GGGGACAAGTTTGTACAAAAAAGCAGGCTTCATGATGGCCGACG AGATCGCCG
attB-SceIF4Eb-R	GGGGACCACTTTGTACAAGAAAGCTGGGTC AACCGTGTAACGGTTCC
attB-SceIF(iso)4E-F	GGGGACAAGTTTGTACAAAAAAGCAGGCTTCATGATGGCGGAGG TCGAGGTTCC
attB-SceIF(iso)4E-R	GGGGACCACTTTGTACAAGAAAGCTGGGTCCCGTCCGCCTCTGCT CG
attB-ScnCBP-F	GGGGACAAGTTTGTACAAAAAAGCAGGCTTCATGATGGAGGCGG CGGTGGAGAA
attB-ScnCBP-R	GGGGACCACTTTGTACAAGAAAGCTGGGTC TCCTCTCAGCCAAGTGT
attB-SCMV-VPg-F	GGGGACAAGTTTGTACAAAAAAGCAGGCTTCATGATGGGAAAGA ACAAGCGCAGCAG
attB-SCMV-VPg-R	GGGGACCACTTTGTACAAGAAAGCTGGGTC GAGACAGGTGTCGCGCACGA
attB-SrMV-VPg-F	GGGGACAAGTTTGTACAAAAAAGCAGGCTTCATGATGGGTAAAA ACAAGAGGAGTAG
attB-SrMV-VPg-R	GGGGACCACTTTGTACAAGAAAGCTGGGTC TTCATGTTCTACTTCAACCT
attB-SCSMV-VPg-F	GGGGACAAGTTTGTACAAAAAAGCAGGCTTCATGATGGGGAAGA AGCGTCGAACTC
attB-SCSMV-VPg-R	GGGGACCACTTTGTACAAGAAAGCTGGGTC CTCACTAGTCAACGCCTGTTG
**Primers used to construct Y2H vectors**	
AD-SceIF4Ea-F	GCCAGTGAATTCCACATGGCCGACGAGATCGACAG
AD-SceIF4Ea-R	TCGATGCCCACCCGGTCAAACCGTGTAACGGTTCC
AD-SceIF4Eb-F	GCCAGTGAATTCCACATGGCCGACGAGATCGCCG
AD-SceIF4Eb-R	TCGATGCCCACCCGGTCAAACCGTGTAACGGTTCC
AD-SceIF(iso)4E-F	GCCAGTGAATTCCACATGGCGGAGGTCGAGGTTCC
AD-SceIF(iso)4E-R	TCGATGCCCACCCGGTTACCGTCCGCCTCTGCTCG
AD-ScnCBP-F	GCCAGTGAATTCCACATGGAGGCGGCGGTGGAGAA
AD-ScnCBP-R	TCGATGCCCACCCGGTTATCCTCTCAGCCAAGTGT
BD-SCMV-VPg-F	ATGGAGGCCGAATTCATGGGAAAGAACAAGCGCAGCAG
BD-SCMV-VPg-R	TCGACGGATCCCCGG GAGACAGGTGTCGCGCACGA
BD-SrMV-VPg-F	ATGGAGGCCGAATTCATGGGTAAAAACAAGAGGAGTAG
BD-SrMV-VPg-R	TCGACGGATCCCCGGTTCATGTTCTACTTCAACCT

BD-SCSMV-VPg-F	ATGGAGGCCGAATTCATGGGGAAGAAGCGTCGAACTC
BD-SCSMV-VPg-R	TCGACGGATCCCCGGCTCACTAGTCAACGCCTGTTG
**Primers used to construct pBridge vectors**	
MCS1-SCMV-VPg-F	GTATCGCCGGAATTCATGGGAAAGAACAAGCGCAGCAG
MCS1-SCMV-VPg-R	TCGACGGATCCCCGGGAGACAGGTGTCGCGCACGA
MCS1-SrMV-VPg-F	GTATCGCCGGAATTCATGGGTAAAAACAAGAGGAGTAG
MCS1-SrMV-VPg-R	TCGACGGATCCCCGGTTCATGTTCTACTTCAACCT
MCS1-SCSMV-VPg-F	GTATCGCCGGAATTCATGGGGAAGAAGCGTCGAACTC
MCS1-SCSMV-VPg-R	TCGACGGATCCCCGGCTCACTAGTCAACGCCTGTTG
MCS2-SCMV-VPg-F	GAAGAGAAAGGTGGCGATGGGAAAGAACAAGCGCAGCAG
MCS2-SCMV-VPg-R	CGGGCTAATGCGGCCGAGACAGGTGTCGCGCACGA
MCS2-SrMV-VPg-F	GAAGAGAAAGGTGGCGATGGGTAAAAACAAGAGGAGTAG
MCS2-SrMV-VPg-R	CGGGCTAATGCGGCCTTCATGTTCTACTTCAACCT
MCS2-SCSMV-VPg-F	GAAGAGAAAGGTGGCGATGGGGAAGAAGCGTCGAACTC
MCS2-SCSMV-VPg-R	CGGGCTAATGCGGCCCTCACTAGTCAACGCCTGTTG
MCS2-SceIF(iso)4E-F	GAAGAGAAAGGTGGCGATGGCGGAGGTCGAGGTTCC
MCS2-SceIF(iso)4E-R	CGGGCTAATGCGGCCTTACCGTCCGCCTCTGCTCG
MCS2-SceIF4Eb-F	GAAGAGAAAGGTGGCGATGGCCGACGAGATCGCCGATGAC
MCS2-SceIF4Eb-R	CGGGCTAATGCGGCCTCAAACCGTGTAACGGTTCCTC
**Primers used for RT-qPCR**	
ScAct1-F	CCTGAAGATCACCCTGTGCT
ScAct1-R	GCAGTCTCCAGCTCCTGTTC
SceIF4Ea/b-F	AAGCATTGGGAAGCAGTGGAAGG
SceIF4Ea/b-R	ACGGTTCCTCGCTCCCTTGTC
SceIF(iso)4E-F	GCAAATGGTGGCAAATGGACTGTC
SceIF(iso)4E-R	CTCTCGCACGGACACTAGCAAC
ScnCBP-F	GGTGCTGGTGCTAGTTGGTGAC
ScnCBP-R	GCCTGATGGTCTGATGCGTTCC
SCMV-CP-F	GGCGAGACTCAGGAGAATACA
SCMV-CP-R	ACCGCCTCCACCAGTAGCTCC
